# Flourishing during stages of substance use recovery among members of The Phoenix: a United States sober-active community

**DOI:** 10.3389/fpubh.2025.1683975

**Published:** 2025-12-09

**Authors:** Katie M. Heinrich, Beth Collinson, Jacquelyn Hillios

**Affiliations:** Department of Research and Evaluation, The Phoenix, Boston, MA, United States

**Keywords:** addiction recovery, flourishing, wellbeing, community, thentest, substance use

## Abstract

**Background:**

Substance use recovery is increasingly understood as a holistically process of personal and social growth rather than abstinence alone. This aligns with the concept of human flourishing, which is understudied in recovery communities. This study aimed to assess self-reported changes in flourishing among members of The Phoenix, a nationwide sober-active community, and to examine whether flourishing differed by recovery stage and length of membership.

**Methods:**

Members in recovery (*N* = 540; 22.8% in early recovery; 45.7% with <1 year of membership) completed a retrospective (“thentest”) cross-sectional survey, rating their flourishing at joining and currently using the 12-item Secure Flourishing Index. Nonparametric analyses examined changes in flourishing and differences by membership length while adjusting for recovery stage.

**Results:**

Members in early recovery reported significantly lower flourishing at joining. Overall flourishing increased by 33.3% from joining to present, with significant improvements across all six subdomains. Members with 5 + years of membership reported greater perceived increases than those with <1 year (*p* < 0.05). Both recovery stage and membership length contributed uniquely to the ANOCOVA, *ƒ* (4,534) = 10.3, *p* < 0.01.

**Discussion:**

Flourishing increased across recovery stages and membership length in The Phoenix community. Findings suggest that participation in recovery communities such as The Phoenix may enhance flourishing, supporting their value as community-based resources for sustained recovery and public health; however longitudinal research is needed.

## Background

Recovery is a dynamic, non-linear process of personal and social growth that can follow multiple pathways (1, 2). Earlier perspectives in the United States (US) often equated recovery as abstinence from substance use; however, contemporary definitions emphasize recovery as a holistic process of rebuilding a meaningful and purposeful life (3, 4). Recent research with over 9,300 US adults in recovery reinforces this broader view, highlighting qualities such as self-honesty, self-responsibility, and balanced living as core aspects of the process (5). Recovery encompasses both intrapersonal growth and social changes (5) and is often facilitated by access to personal, social and community resources—or recovery capital (6). These evolving definitions reflect a shift from a narrow focus on abstinence toward a strengths-based and multidimensional understanding of recovery in the US.

The additive process of recovery characterized by growth and strengthened capabilities, helps lead toward flourishing (7), which is “a state of complete physical, social, emotional, cognitive, volitional, and spiritual wellbeing” (8). Yet, this process is not straightforward as shifting life circumstances and social environments contribute to recovery being non-linear and subject to ongoing changes (9). This fluidity highlights the importance of adaptability and support in sustaining long-term recovery. Although flourishing as an integrative construct has rarely been studied in relation to substance use disorders, many of its domains, such as social connectedness, meaning and purpose in life, and emotional wellbeing, are well established as protective factors against addiction and related harms. For example, lack of supportive relationships, low life satisfaction, and diminished meaning and purpose are consistently linked to greater risk of substance use and relapse (10), whereas higher levels of subjective wellbeing and purpose are associated with reduced likelihood of initiating or continuing use (11, 12). Flourishing has also been linked more broadly to reduced engagement in risky or unhealthy behaviors, though causal data are needed (13). Together, these findings suggest flourishing may play a critical role in both preventing substance use and sustaining recovery, yet it has not been extensively examined within recovery communities.

Within recovery community, flourishing and its relationship to mental health and substance use outcomes remains underexplored. Many of the domains that constitute flourishing (e.g., meaning and purpose, social relationship, and material or financial stability) (14) are embedded within the concept of recovery capital and have long been recognized as supporting sustained recovery (15). Recovery processes often foster gains in these areas, yet flourishing provides an integrative framework for understanding how these domains combine to shape overall well-being and the likelihood that “all aspects of a person’s life are good” (16). Efforts to study these dynamics are further complicated by the use of diverse measurement scales, which limits comparability across studies (17).

The Global Flourishing Study, covering 22 countries and territories around the world, is assessing human flourishing across domains including happiness and life satisfaction, mental and physical health, meaning and purpose, character and virtue, close social relationships, and financial and material stability (18). Notably, these domains of flourishing as captured in VanderWeele’s index (16) are strongly aligned with contemporary definitions of recovery (19). Building on these perspectives, this study considers flourishing as a framework for understanding recovery, positioning it as a multidimensional construct that encompasses both healing and growth.

The Phoenix is a national nonprofit organization in the US that provides free, peer-led, substance-free activities to support individuals in recovery, those seeking to reduce their use, and allies. Participation is open to anyone with at least 48 h of sobriety from alcohol or non-prescription drugs, and programming spans yoga, fitness, hiking, music, art, and other collective activities delivered both in-person and virtually across the United States. Rooted in the fabric of local communities, The Phoenix promotes engagement through shared interests and enjoyable experiences, rather than activities solely focused on recovery processes.

Peer support is widely recognized as a cornerstone of recovery-oriented systems of care. Across the continuum, individuals with lived experience provide support by inspiring hope, sharing stories, participating in mutual-aid groups, or working as trained recovery coaches (20). While powerful, these approaches are often structured around explicitly “recovery-centric” conversations and, in the case of coaching, one-to-one relationships.

The Phoenix represents a distinct model, where “peer-led” means that activities are designed and facilitated by volunteers who share a personal connection to the mission, whether through their own recovery, the experience of a loved one, or allyship. While Phoenix volunteers also share stories and provide encouragement, their primary role is to create inclusive, substance-free environments where recovery support emerges naturally through group activities. Volunteers are empowered to shape the calendar of offerings based on their own skills and passions, supported by Phoenix’s infrastructure and technology. This “people helping people” ethos extends beyond formal volunteer roles to the broader community, where members provide support for one another (21). Unlike time-limited or clinically focused services, The Phoenix provides ongoing opportunities for connection, empowerment, and wellbeing. Evidence shows that participation in The Phoenix increases connection, hope, empowerment, and health (21–23). However, its impact on flourishing has not yet been studied.

This study aimed to examine self-reported changes in flourishing among members of The Phoenix, exploring differences by recovery stage and length of membership. We hypothesized that those newer to recovery would report lower flourishing when joining The Phoenix, but that groups at all stages of recovery would report increased flourishing from joining to currently. We also expected longer Phoenix membership to be associated with greater increases in flourishing.

## Methods

### Design

This study employed a retrospective (“thentest”) cross-sectional within-person survey design (24, 25). Members were first asked to recall their initial feelings upon joining The Phoenix and respond to survey items reflecting how they felt “then.” They were then asked to provide responses for how they felt “currently.” Whereas change is often assessed through pretest-posttest comparisons (25), a retrospective pretest (i.e., thentest) allows researchers to capture shifts in internal standards – commonly referred to as response shift or recalibration (26, 27). In this design, the effect of time is operationalized as the difference between current and thentest responses (24). Although the thentest approach may introduce recall bias (24, 25), it also offers an introspective means for members to evaluate perceived changes in themselves over time and has been found to provide more realistic perceptions in hindsight (27). The study received ethics approval from NDRI-USA and all participants completed informed consent online, prior to starting a survey.

### Participants

Up to three emails, including two reminders, were sent to 10,000 Phoenix members who had opted in to receive email communication, with a request to complete an online survey between March–May, 2023. After receiving 985 responses and meeting our recruitment goal, we closed the survey. For this paper, we focused on the 740 members who were in recovery (75% of respondents); 540 of those had complete data for flourishing and were included in the analysis. Over half (50.5%) were female, followed by male (45.7%), or other genders (2.7%; e.g., nonbinary, transgender), with 9 participants preferring not to answer (1.2%). The majority were white (81.7%), followed by multiple races (3.2%), Alaska Native/American Indian (1.9%), black/African American (1.8%), and Asian (1.2%), among others. Most were heterosexual (76.3%), followed by bisexual (7.7%), gay (6.1%), and lesbian (3.5%). Average age was 45.7 ± 11.2 years; 22.8% were in early recovery (<1 year), 36.1% were in sustained recovery (1–5 years), and 41.1% were in stable recovery (>5 years). Most had been Phoenix members either <1 year (45.7%) or 1–5 years (43.3%), with some having over 10 years of membership (4.1%).

### Measures

After clicking an emailed survey link, members completed informed consent. They provided demographic information (i.e., gender, race/ethnicity, sexual orientation, age), followed by length of recovery and membership duration with The Phoenix, both in years. They completed the 12-item Secure Flourishing Index (16) for how they felt when they first joined The Phoenix (i.e., “then”), and a second time for currently (i.e., “now”) across six domains: life satisfaction and happiness, mental and physical health, meaning and purpose, character and virtue, social relationships, and material and financial security, with two items per domain. Items were answered on a scale from 0 (low) to 10 (high). Previous research has shown strong internal consistency with Cronbach’s alpha >0.76 across samples including healthcare workers and international adults (28, 29) and acceptable discriminant validity between domains (30). The “thentest” method was used to assess perceived changes from then to now (25).

Self-reported recovery status was categorized according to the Betty Ford Institute’s categories of early recovery (1–11 months), sustained recovery (1 to <5 years), and stable recovery (5 + years) (31).

### Analysis

Data were imported into SPSS v. 30 for analysis. Since the flourishing data failed the Shapiro–Wilk test of normality, non-parametric tests were used. Kruskal-Wallis H tests were used for comparisons between groups, and Mann–Whitney U tests for follow-up to determine paired differences. Wilcoxon signed-rank tests were used for differences in flourishing scores. To explore differences by length of Phoenix membership while controlling for recovery status, we used rank-based ANCOVA (32) on the change score for secure flourishing (thentest minus current average). This nonparametric approach reduces the influence of skewed distributions and outliers by rank-transforming the dependent variable, while still allowing adjustment for covariates (32).

## Results

### Flourishing scores

At first joining The Phoenix, flourishing scores differed significantly by stage of recovery, *H* = 69.45, *p* < 0.001 (see Table 1). Members in early recovery reported the lowest flourishing scores, followed by those in sustained recovery, as compared to those in stable recovery. Flourishing scores did not significantly differ thinking back to joining by current length of Phoenix membership.

**Table 1:** Flourishing scores at joining by stage of recovery and current length of Phoenix membership (Median ± IQR).

Subgroups	Happiness & life satisfaction	Mental & physical health	Meaning & purpose	Character & virtue	Close social relationships	Financial & material stability	Flourishing score
Early recovery	4.5 ± 3.5^a^^,^^b^	4.0 ± 3.1^a^^,^^b^	4.0 ± 4.0^a^^,^^b^	5.0 ± 3.6^a^^,^^b^	3.0 ± 3.1^a^^,^^b^	5.5 ± 5.0^a^^,^^b^	4.4 ± 2.8^a^^,^^b^
Sustained recovery	5.5 ± 3.4^b^	5.0 ± 3.5^b^	5.0 ± 4.0^b^	5.5 ± 3.0^b^	4.5 ± 3.4^b^	6.0 ± 4.5^b^	5.3 ± 2.8^b^
Stable recovery	6.5 ± 3.0	6.5 ± 3.5	7.0 ± 3.5	7.0 ± 3.0	6.0 ± 3.5	7.0 ± 5.0	6.4 ± 2.8
<1 year Phoenix member	5.0 ± 3.5	5.5 ± 3.5	5.5 ± 4.5	6.0 ± 3.5	4.5 ± 4.0	5.8 ± 5.0	5.4 ± 2.2
1 to <5 years Phoenix member	6.0 ± 3.5	6.0 ± 3.5	5.5 ± 3.5	6.0 ± 3.4	5.0 ± 4.0	6.5 ± 4.5	5.6 ± 2.1
5–9 years Phoenix member	5.5 ± 3.3	5.0 ± 4.0	5.8 ± 4.4	6.0 ± 3.4	4.5 ± 3.3	6.0 ± 4.9	5.5 ± 2.2
10 + years Phoenix member	5.0 ± 3.0	6.0 ± 3.5	5.0 ± 4.0	5.8 ± 4.5	4.5 ± 4.5	5.0 ± 4.0	5.0 ± 2.0
Total sample	6.0 ± 3.0	5.5 ± 3.5	5.5 ± 4.5	6.0 ± 3.5	5.0 ± 3.5	6.0 ± 5.0	5.5 ± 3.2

a Significantly lower than sustained recovery (*p*-values ranged from <0.001 to <0.05).

b Significantly lower than stable recovery (*p*-values ranged from <0.001 to <0.01).

Table 2 displays current flourishing scores. Scores differed significantly by stage of recovery, *H* = 47.28, *p* < 0.001. There was a significant difference in current flourishing by length of Phoenix membership, *H* = 20.43, *p* < 0.001. A Mann–Whitney U test found that those with <1 year of Phoenix membership reported significantly lower flourishing than those with 1 + years of membership (*U* = 22,861.5, *Z* = 3.96, *p* < 0.001) and those with 5–9 years of membership (*U* = 3,156.5, *Z* = 2.81, *p* < 0.05).

**Table 2:** Current flourishing scores by stage of recovery and current length of Phoenix membership (Median ± IQR).

Subgroups	Happiness & life satisfaction	Mental & physical health	Meaning & purpose	Character & virtue	Close social relationships	Financial & material stability	Flourishing score
Early recovery	7.0 ± 3.0^a^^,^^b^	6.8 ± 3.0^a^^,^^b^	6.8 ± 3.0^a^^,^^b^	7.0 ± 2.5^a^^,^^b^	6.5 ± 4.0^a^^,^^b^	6.0 ± 4.0^a^^,^^b^	6.5 ± 2.8^a^^,^^b^
Sustained recovery	8.0 ± 2.0	7.5 ± 2.0	7.5 ± 3.0^c^	8.0 ± 2.0^c^	7.5 ± 2.5	8.0 ± 4.0^c^	7.4 ± 2.0^c^
Stable recovery	8.0 ± 2.0	7.5 ± 1.5	8.0 ± 2.0	8.0 ± 2.0	7.5 ± 3.0	8.5 ± 3.0	7.8 ± 1.8
<1 year Phoenix member	7.5 ± 2.0^d^^,^^e^	7.0 ± 2.0^d^	7.5 ± 3.0^d^^,^^e^	7.5 ± 2.5^d^^–^^f^	7.0 ± 3.0^d^^–^^f^	7.0 ± 4.5^d^	7.1 ± 2.2^d^^–^^f^
1 to <5 years Phoenix member	8.0 ± 2.0	7.5 ± 1.5	8.0 ± 2.5	8.0 ± 2.0	7.5 ± 3.0	8.5 ± 3.3	7.7 ± 1.8
5–9 years Phoenix member	8.0 ± 2.0	7.8 ± 2.8	8.5 ± 3.0	8.0 ± 1.5	8.0 ± 2.9	7.8 ± 2.0	7.8 ± 2.0
10 + years Phoenix member	8.0 ± 1.6	7.8 ± 2.3	7.5 ± 2.6	8.3 ± 2.1	7.8 ± 3.1	8.3 ± 4.0	7.8 ± 2.0
Total sample	7.5 ± 2.0	7.5 ± 2.0	7.5 ± 3.0	8.0 ± 2.5	7.0 ± 3.0	8.0 ± 4.5	7.5 ± 2.1

a Significantly lower than sustained recovery at *p* < 0.001.

b Significantly lower than stable recovery at *p* < 0.001.

c Significantly lower than stable recovery at *p* < 0.05.

d Significantly lower than 1 to <5 years (*p*-values ranged from <0.001 to <0.01).

e Significantly lower than 5–9 years (*p*-values ranged from <0.05 to <0.01).

f Significantly lower than 10 + years (*p* < 0.05).

### Differences in flourishing

For the entire sample, flourishing increased 33.3% from joining to currently, with total scores increasing from 5.4 to 7.2; *Z* = −17.37, *p* < 0.001. Increases were also statistically significant across each flourishing subdomain: happiness and life satisfaction (*Z* = −15.40, *p* < 0.001), mental and physical health (*Z* = −15.36, *p* < 0.001), meaning and purpose (*Z* = −15.22, *p* < 0.001), character and virtue (*Z* = −16.15, *p* < 0.001), close social relationships (*Z* = −15.91, *p* < 0.001), and financial and material stability (*Z* = −9.47, *p* < 0.001).

Increases in flourishing differed significantly by stage of recovery (*H* = 18.88, *p* < 0.001). Those in early recovery (Median ± IQR = 1.5 ± 2.8; *U* = 11,261.0, *Z* = 2.70, *p* = 0.007) and those in sustained recovery (Median ± IQR = 1.8 ± 2.8; *U* = 16,465.0, *Z* = 4.22, *p* < 0.001) reported significantly greater increases in flourishing than those in stable recovery (Median ± IQR = 0.9 ± 2.0).

Flourishing also significantly differed by length of Phoenix membership (*H* = 12.629, *p* = 0.006). Median (± IQR) improvements in flourishing scores increased with longer Phoenix membership; <1 year = 1.1 ± 2.3, 1 to < 5 years = 1.5 ± 2.3, 5–9 years = 2.3 ± 3.1, 10 + years = 2.5 ± 1.2. Mann–Whitney U tests found significant differences in flourishing increases where those with <1 year of membership were significantly lower than those with 5–9 years (*U* = 3,161.0, *Z* = 2.80, *p* = 0.005) and those with 10 + years of membership (*U* = 1,912.5, Z = 2.30, *p* = 0.021).

Results of the rank-based ANCOVA found a significant overall model, *ƒ* (4, 534) = 10.3, *p* < 0.01, with both recovery stage *ƒ* (1, 534) = 27.68, *p* < 0.01, *η^2^* = 0.049, and length of Phoenix membership, *ƒ* (3, 534) = 9.91, *p* < 0.01, *η^2^* = 0.053, contributing unique variance. Adjusted means indicated that those with longer Phoenix membership (5–9 years, *M* = 357.5; 10 + years, *M* = 365.1) had significantly greater ranked flourishing differences compared to those with less than 1 year of membership (*M* = 236.3). Phoenix membership of 1–5 years (*M* = 284.4) was also associated with greater differences in flourishing than those with less than 1 year but did not differ significantly from the 10 + years of membership group.

## Discussion

In this study of self-reported flourishing changes among members of The Phoenix, a national nonprofit providing free, peer-led, substance-free activities to support individuals in recovery, those seeking to reduce their use, and allies, we found significant perceived increases in flourishing from at joining to currently. As hypothesized, members newer to recovery reported significantly lower flourishing scores at joining. As well, those in sustained recovery also reported significantly lower flourishing scores at joining than those in stable recovery. These patterns reflect prior research describing recovery as a dynamic and staged process in which well-being improves, along with increases in recovery capital and social connectedness (33). All recovery stages significantly increased in flourishing as hypothesized, although increases were significantly greater for those in early and sustained recovery. Also as hypothesized, those with 5–9 years and 10 + years of Phoenix membership had significantly greater increases in flourishing than members of <1 year. Findings suggest that longer membership with The Phoenix is associated with larger perceived increases in flourishing, even after accounting for differences in length of recovery, underscoring the importance of considering individual and contextual factors simultaneously.

Our study findings reinforce the view of recovery as a process of personal and social growth rather than symptom reduction alone (5, 7), which seems to complement flourishing (7). Our flourishing scores (mean = 7.22 for the first five subdomains) were nearly identical to those reported for the US general population (mean = 7.18) (18). This suggests that members who sustain engagement with The Phoenix achieve levels of well-being comparable to non-clinical populations.

Importantly, each flourishing subdomain increased significantly across recovery stages and membership lengths, suggesting that gains were multidimensional rather than confined to a single domain. This pattern is consistent with research emphasizing the interdependence of personal, social, and community recovery capital in promoting overall flourishing (5, 15). This provides early evidence that The Phoenix’s community-based approach—rooted in peer-support, community, and meaningful activities —appears to support holistic growth, complementing previous research linking physical activity and peer support to enhanced mental health and purpose in recovery (34).

Current levels of flourishing varied across recovery stages in a manner consistent with the non-linear nature of the recovery process (1, 2, 9). Individuals in sustained recovery often reported flourishing levels comparable to those in stable recovery, whereas individuals in early recovery consistently reported lower levels. These differences underscore the importance of community engagement in bridging early deficits in recovery capital, a finding consistent with, but extending prior models by demonstrating holistic growth across all flourishing domains (15).

Despite consensus that flourishing is a multidimensional construct, there are multiple ways it has been measured (17). We used the Secure Flourishing Index (16), a globally validated measure currently featured in international surveillance efforts (18). By applying this measure in a recovery community context, our study extends the use of flourishing metrics beyond general and occupational populations to individuals in substance use recovery who are underrepresented in flourishing research. In addition to rich comparisons, this opens opportunities for longitudinal monitoring and for comparing recovery community outcomes with global benchmarks of human flourishing.

Taken together, our findings converge with and extend previous work on recovery capital, peer-based supports, and holistic wellbeing. They support the potential of community-based models such as The Phoenix to foster flourishing and underscore the need for longitudinal, cross-context studies to clarify temporal and causal pathways.

To our knowledge, this is the first study to use the Secure Flourishing Index in a recovery community context. Strengths include the use of a large, national sample that enabled sub-group analyses by both recovery stage and length of Phoenix membership, as well as the use of a retrospective (“thentest” pre-post design), which may have reduced social-desirability bias (27). However, several limitations should be noted. The retrospective cross-sectional design introduces potential selection bias among those who chose to participate, as well as survivor bias where those successful in recovery were likely to continue membership and receive a survey. Recall and self-serving bias are also possible when participants evaluate prior states of flourishing. The absence of a control or comparison group makes it impossible to rule out changes occurring due to maturation, regression to the mean, or other factors. In addition, information on household income, socioeconomic status and substance use or treatment histories was not collected, constraining interpretation of contextual factors associated with flourishing. Finally, while rank-based ANCOVA addressed non-normal data distributions, results should be Interpreted in terms of ranked rather than raw changes, which may limit comparability across studies. Future research should employ longitudinal designs and incorporate broader demographic and clinical variables to further examine the mechanisms linked recovery community engagement and flourishing.

## Conclusion

This study examined whether flourishing differed by recovery stage and length of membership among participants in The Phoenix, a nationwide sober-active community in the US. Consistent with our hypotheses, flourishing was perceived to increase from joining to the present, with larger increases among members in early and sustained recovery and among those with longer membership duration. These findings suggest that community engagement contributed uniquely to flourishing beyond time in recovery alone.

Flourishing within recovery may be a socially dynamic process supported by connection, meaningful engagement, and opportunities for contribution rather than an individual achievement. Positioning flourishing as a measurable recovery outcome broadens the understanding of recovery beyond abstinence alone, and emphasizes dignity, purpose, and participation in life. Findings underscore the importance of investing in community-based recovery environments as public health assets and support future research on how social connection and purpose-driven engagement promote flourishing across diverse recovery populations. Future longitudinal research should examine how community-based recovery environments foster flourishing over time and how broader structural factors, such as poverty, stigma, and access to care, influence individuals’ capacity to thrive.

## Data Availability

The raw data supporting the conclusions of this article will be made available by the authors, without undue reservation.
